# High-flow oxygen via nasal cannulae in patients with acute hypoxemic respiratory failure: a systematic review and meta-analysis

**DOI:** 10.1186/s13643-017-0593-5

**Published:** 2017-10-16

**Authors:** Murdoch Leeies, Eric Flynn, Alexis F. Turgeon, Bojan Paunovic, Hal Loewen, Rasheda Rabbani, Ahmed M. Abou-Setta, Niall D. Ferguson, Ryan Zarychanski

**Affiliations:** 1 Department of Emergency Medicine, University of Manitoba, JJ399-700 William Avenue, Ann Thomas Building, Winnipeg, MB R3E0Z3 Canada; 2 Department of Internal Medicine, Section of Critical Care, University of Manitoba, Winnipeg, MB Canada; 3 Department of Community Health Sciences, University of Manitoba, Winnipeg, MB Canada; 4 Division of Critical Care Medicine, Department of Anesthesiology, Université Laval, Québec City, Québec Canada; 5 Neil John Mclean Library, University of Manitoba, Winnipeg, MB Canada; 6 George & Fay Yee Center for Healthcare Innovation, University of Manitoba/Winnipeg Regional Health Authority, Winnipeg, MB Canada; 7 Department of Medicine, Division of Respirology, University Health Network and Mount Sinai Hospital, Toronto General Research Institute, Interdepartmental Division of Critical Care Medicine, Toronto, ON Canada; 8 Departments of Medicine and Physiology, and Institute of Health Policy, Management and Evaluation, University of Toronto, Toronto, ON Canada

**Keywords:** High flow, Nasal cannula, Oxygen therapy, Respiratory failure, Acute respiratory failure, Hypoxemic respiratory failure

## Abstract

**Background:**

We performed a systematic review and meta-analysis to evaluate the efficacy and safety of high-flow oxygen via nasal cannulae (HFNC) compared to non-invasive ventilation (NIV) and/or standard oxygen in patients with acute, hypoxemic respiratory failure.

**Methods:**

We reviewed randomized controlled trials from CENTRAL, EMBASE, MEDLINE, Scopus and the International Clinical Trials Registry Platform (inception to February 2016), conference proceedings, and relevant article reference lists. Two reviewers independently screened and extracted trial-level data from trials investigating HFNC in patients with acute, hypoxemic respiratory failure. Internal validity was assessed in duplicate using the Cochrane Risk of Bias tool. The strength of evidence was assessed in duplicate using the Grading of Recommendations Assessment, Development and Evaluation framework. Our primary outcome was mortality. Secondary outcomes included dyspnea, PaO_2_:FiO_2_ ratio, PaCO_2_, and pH. Safety outcomes included respiratory arrest, intubation, delirium, and skin breakdown.

**Results:**

From 2023 screened citations, we identified seven trials (1771 patients) meeting inclusion criteria. All trials were at high risk of bias due to lack of blinding. There was no evidence for a mortality difference in patients receiving HFNC vs. NIV and/or standard oxygen (RR 1.01, 95% CI 0.69 to 1.48, *I* ^2^ = 63%, five trials, 1629 patients). In subgroup analyses of HFNC compared to NIV or standard oxygen individually, mortality differences were not observed. Measures of patient tolerability were heterogeneous. The PaO_2_:FiO_2_ ratio at 6–12 h was significantly lower in patients receiving oxygen via HFNC compared to NIV or standard oxygen for hypoxemic respiratory failure (MD − 53.34, 95% CI − 71.95 to − 34.72, *I* ^2^ = 61%, 1143 patients). There were no differences in pH, PaCO_2_, or rates of intubation or cardio-respiratory arrest. Delirium and skin breakdown were infrequently reported in included trials.

**Conclusions:**

In patients with acute hypoxemic respiratory failure HFNC was not associated with a difference in mortality compared to NIV or standard oxygen. Secondary outcomes including dyspnea, tolerance, and safety were not systematically reported. Residual heterogeneity and variable reporting of secondary outcomes limit the conclusions that can be made in this review. Prospective trials designed to evaluate the efficacy and safety of HFNC in patients with acute hypoxemic respiratory failure are required.

**Electronic supplementary material:**

The online version of this article (10.1186/s13643-017-0593-5) contains supplementary material, which is available to authorized users.

## Background

Acute hypoxemic respiratory failure is widely prevalent in acutely ill patients. Supplemental oxygen therapy is administered via nasal prong, facemask, non-invasive, or invasive ventilation modalities to correct hypoxemia. Standard nasal prong or facemask systems are limited in the fraction of inspired oxygen, gas flow rate, airway pressure, and comfort delivered as compared to other modalities [1]. Traditional non-invasive positive pressure ventilation via facemask has been applied with benefit to heterogeneous populations with acute respiratory failure, including those with chronic obstructive pulmonary disease [2], cardiogenic pulmonary edema [3], and as a weaning strategy in adults intubated for acute respiratory failure [4]. In a systematic review of non-invasive positive pressure ventilation in patients with acute hypoxemic respiratory failure; however, there was no significant reduction in in-hospital mortality [5]. The need for invasive ventilation in patients with acute hypoxemic respiratory failure represents a final common pathway when all other oxygen delivery systems are inadequate and is associated with significant morbidity and mortality [6, 7].

High-flow oxygen via nasal cannula is a non-invasive therapy where heated, humidified oxygen is delivered via large-bore nasal cannula at flow rates up to 60 L/min. The fraction of inspired oxygen can be titrated to 100%, and the mean airway pressure increases with increasing gas flow rates [8, 9]. Observational studies suggest that high-flow oxygen via nasal cannulae is associated with improved oxygenation, decreased respiratory rate, increased lung volumes, and improved patient comfort as compared to standard oxygen therapy [10–14] and may be better tolerated than non-invasive ventilation [15]. A recent retrospective analysis of patients initially treated with high-flow oxygen via nasal cannulae but ultimately requiring endotracheal intubation, however, demonstrated lower rates of successful extubation, fewer ventilator-free days, and higher ICU mortality in those with longer durations (> 48 h) of high-flow nasal oxygen prior to intubation [16]. The efficacy and safety of high-flow oxygen via nasal cannula for *acute hypoxemic respiratory failure* in randomized trials remains uncertain.

The objective of this systematic review was to identify, critically appraise, and meta-analyze data from prospective randomized trials comparing high-flow oxygen via nasal cannula to non-invasive ventilation and/or standard oxygen therapy in adult patients with acute hypoxemic respiratory failure.

## Methods

We conducted a systematic review adherent to the Methodological Expectations of Cochrane Intervention Reviews framework [17]. Reporting was consistent with the criteria outlined in the Preferred Reporting Items for Systematic Reviews and Meta-Analyses guidelines [18]. This review was ineligible for registration in PROSPERO as the research was too far underway by the time of potential registration. A full protocol for this research can be found in Additional file 1. Ethics approval was not required for this meta-analysis.

### Populations, interventions, comparators, outcome measures, settings, and study designs

We posed the question “In adults admitted to an emergency department (ED) or an intensive care unit (ICU) with acute hypoxemic respiratory failure, what is the efficacy and safety of high-flow oxygen via nasal cannula compared to non-invasive ventilation or standard oxygen on mortality, incidence of intubation, patient tolerability and adverse events?” Prospective, randomized controlled trials of adult patients with author-defined acute hypoxemic respiratory failure (at least 80% of the study population) receiving high-flow, humidified oxygen via nasal cannulae were included.

We excluded trials where high-flow oxygen via nasal cannulae was applied prophylactically. We also excluded cross-over trials due to potential carry-over effects of the interventions. Our primary outcome measure was the incidence of mortality at longest duration of follow-up. Secondary outcomes were incidence of endotracheal intubation, patient tolerability, dyspnea rating, and physiologic variables (PaO_2_:FiO_2_ ratio, PaCO_2_, pH). Safety outcomes were cardio-respiratory arrest, delirium, and skin breakdown. All outcomes were extracted at the longest duration of study follow-up. The roles of each systematic review team member are presented in Additional file 2.

### Search strategy for identification of studies

We searched MEDLINE (Ovid), EMBASE (Ovid), and CENTRAL (the Cochrane Library–Wiley) from inception to February 2016 using individualized search strategies prepared for each database. We performed a forward search in Scopus and Web of Science to identify additional relevant citations. In order to identify ongoing, planned, or completed but not yet published trials, we searched the World Health Organization’s International Clinical Trials Registry Platform. The search strategy for MEDLINE is presented in Additional file 3. Conference abstracts were searched electronically and reviewed, in duplicate, by the same investigators who screened and extracted data from primary studies (ML, EF). We searched abstracts and conference proceedings for the following societies (2011–2016): *American College of Emergency Physicians*, *American Thoracic Society*, *Canadian Association of Emergency Physicians*, *Canadian Critical Care Society*, *European Society of Intensive Care Medicine*, *and Society of Critical Care Medicine*. There was no language restriction employed. Our electronic search strategy was peer-reviewed according to the Canadian Agency for Drugs and Technologies in Health recommendations [19]. The reference lists of relevant narrative and systematic reviews as well as all included trials were hand-searched for possible relevant citations. Reference Management was performed using EndNote™ (ver. X7.5, Thompson Reuters, USA).

We employed a multi-step process for study selection. Initially, two reviewers (M.L, E.F.) independently screened the titles and abstracts of search results to determine whether each study met inclusion criteria. Each report was classified as *include*, *exclude*, *unclear*, or *duplicate of another citation*. The full text of all reports classified as include or unclear by either reviewer were retrieved for formal review. Next, the two reviewers independently assessed the full text of each trial report, employing a standardized screening form that outlined the predetermined inclusion and exclusion criteria. The form was pilot tested on a sample of studies. All disagreements between the two primary reviewers were resolved by consensus.

### Data abstraction

Data were abstracted using a standardized, piloted form and entered into a Microsoft Excel™ database (Microsoft Corp., Redmond, WA). Two reviewers (M.L, E.F.) independently extracted data from individual trial reports, with disagreements resolved through consensus. The following data were extracted from each study where available: author identification, year of publication, language of publication, source of study funding, study design, study population, patient characteristics (age, sex, SAPS II score or other severity of illness score, etiology of respiratory *failure*), *intervention* (e.g., method of oxygen administration) and its *comparator*, as well as results reported for the outcomes of interest.

### Internal and external validity assessments

We assessed the internal validity of included trials in duplicate using the Cochrane Collaboration Risk of Bias tool [20, 21]. This tool includes six domains (sequence generation, allocation concealment, blinding, incomplete outcome data, selective outcome reporting, and “other” sources of bias) and classifies the overall risk of bias. If one or more individual domains are assessed as having a high risk of bias, the trial is rated as having a high risk of bias. All domains must have been rated as having a low risk of bias for the overall risk of bias to be classified as low. In cases of unclear risk of bias or mixed assessments of low and unclear risk of bias, the overall score was classified as having an unclear risk of bias. Information regarding methodological quality was used to guide sensitivity analyses and explore sources of heterogeneity.

We applied the Grading of Recommendations Assessment, Development and Evaluation (GRADE) approach in order to grade the strength of the evidence for our primary outcome [22]. Two reviewers (M.L., R.Z.) evaluated the strength of the body of evidence individually with discrepancies resolved through consensus. Assessment domains include risk of bias, inconsistency, indirectness, imprecision, and other factors. The strength of evidence is classified as “high,” “moderate,” “low,” or “very low.”

### Measures of treatment effect

We analyzed data from included trials using Review Manager (RevMan version 5.3.5, The Nordic Cochrane Centre, The Cochrane Collaboration, Copenhagen, Denmark). We expressed pooled continuous data as mean differences with 95% confidence intervals. Pooled dichotomous data were presented as risk ratios with 95% confidence intervals. We used random-effects models for all analyses. We quantified statistical heterogeneity of the data using the I-squared test with 95% uncertainty intervals [23]. Trial outcomes and summary effect measures were reported based on intention-to-treat data. Publication bias could not be statistically assessed due to the low number of included trials. All tests of statistical inference reflect a two-sided *α* of 0.05.

### Subgroup analysis

A priori specified subgroup analyses for our primary outcome of mortality included cardiac surgical vs. general ICU patients (population), duration of study therapy (timing), and non-invasive ventilation vs. standard oxygen therapy (comparator) (Additional file 1). Post hoc subgroup analyses included the iterative exclusion of each individual trial to evaluate for individual study effects.

### Trial sequential analysis

Many rigorous meta-analyses lack the statistical power to estimate intervention effects, and the spurious rejection of a true null hypothesis (type I error) or acceptance of a false null hypothesis (type II error) can lead to incorrect conclusions regarding intervention effects [24]. Trial sequential analysis is a frequentist method to control type I and type II errors in meta-analyses that calculates a required information size and clarifies whether additional trials are required [25]. In response to reviewer recommendations, we performed a post hoc trial sequential analysis for the primary outcome mortality.

## Results

**Fig. 1:**
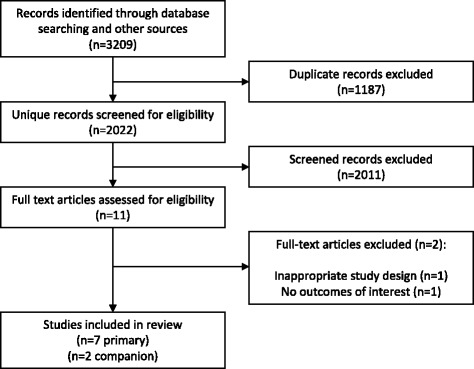
PRISMA flow diagram

**Fig. 2:**
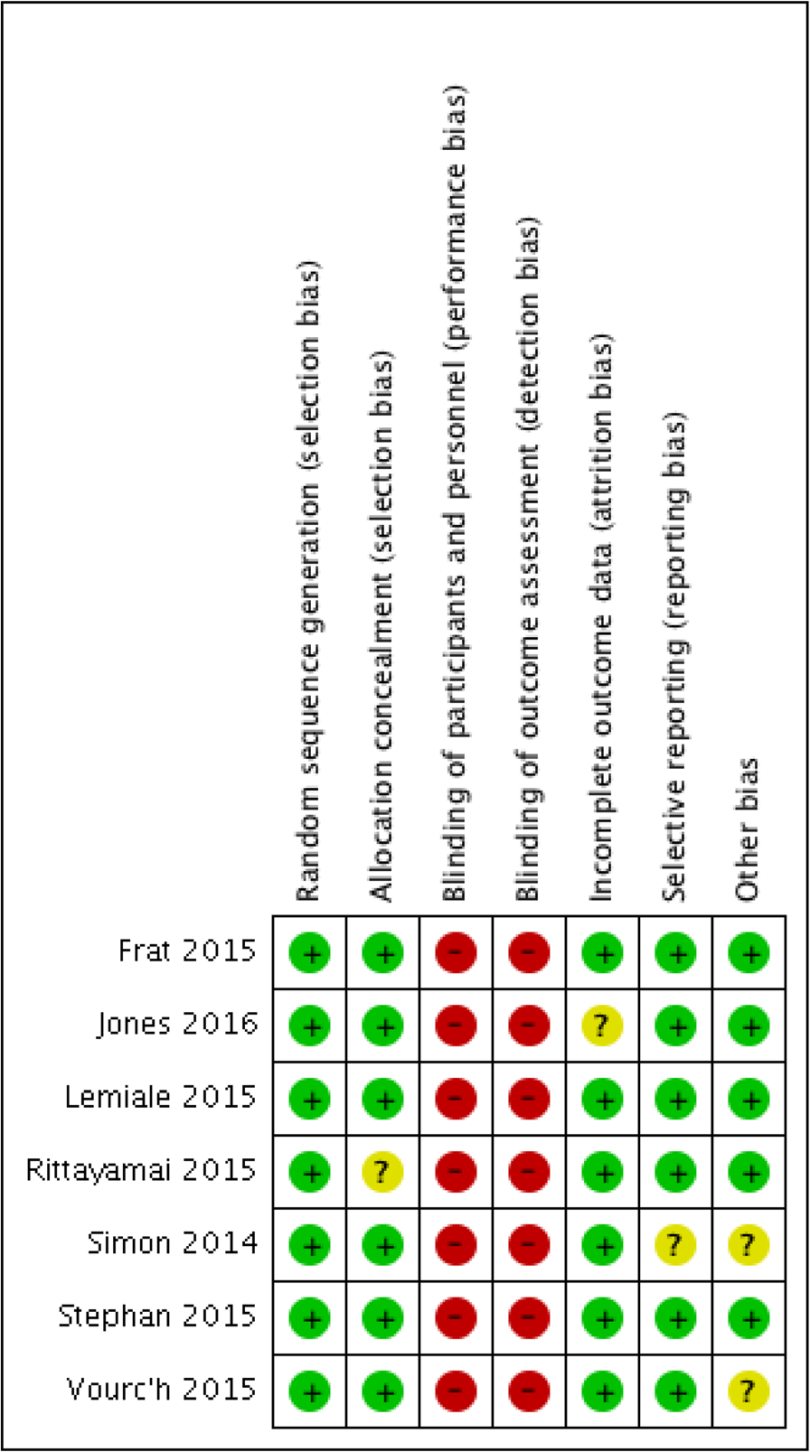
Risk of bias summary

Of the 3209 citations identified from electronic and hand-searches, we included seven unique trial reports [26–31] (plus two companion publications [32, 33]) that enrolled 1771 patients (range 40 to 830) (Table 1; Fig. 1). Publication year ranged from 2014 to 2016. The mean age of enrolled patients in individual trials ranged from 49 to 75 years. The majority of participants were men (62 and 65%, high-flow nasal oxygen and comparators, respectively). Of the seven included trials four were multicenter [26–29]. Five trials were conducted in European centers in France, Belgium, and Germany [26–29, 31], one in New Zealand [34], and one in Thailand [30]. All trials were designed as parallel group randomized controlled trials with 1 designed as a non-inferiority trial [27]. All trials were published in English-language journals. All trials were classified as having an overall high risk of bias due to lack of blinding of patients and personnel (Fig. 2). Settings included mixed medical surgical ICUs [26, 28, 29, 31], cardiac surgical ICUs [27], and emergency departments [30, 34]. The etiology of acute respiratory failure was heterogeneously reported.

**Table 1:** Characteristics of included trials

Trial	Total (*n*), HFNC/control	Setting	Severity of illness score	Intervention protocol	Control protocol	Protocol duration	Longest follow-up
Frat 2015 [26]	106/207	Medical and surgical ICU	SAPS II; HFNC 25 ± 9, standard 24 ± 9, NIV 27 ± 9	HFNC	Standard oxygen arm and NIV arm	Recovery or intubation	90 days
Jones 2016 [34]	172/150	Emergency Department	NR	HFNC	standard oxygen	Recovery or admission	90 days
Lemiale 2015 [29]	53/49	Immuno-compromised ICU	SAPS II; HFNC 42 (29.5–52), control 37.5 (31.5–46.5)	HFNC	Standard oxygen venturi mask	120 min	ICU stay
Rittayamai 2015 [30]	20/20	Emergency department	NR	HFNC	Standard oxygen	ED stay	60 min
Simon 2014 [31]	20/20	Medical and surgical ICU	SAPS II; HFNC 43 ± 13, control 46 ± 10	HFNC	NIV	15 min pre-, 50 min post-bronchoscopy	28 days
Stephan 2015 [27]	414/416	Post-cardiac surgery ICU	SAPS II; HFNC 29 (27.8–30.1), control 28.8 (27.7–30.0)	HFNC	NIV	NR	ICU stay
Vourc’h 2015 [28]	63/61	Medical and surgical ICU	SAPS II; HFNC 54.5 ± 20.2, control 51.3 ± 16.5	HFNC	Face mask	4 min +	28 days

*HFNC* high-flow nasal cannulae, *NR* not reported, *ICU* intensive care unit, *ED* emergency department, *NIV* non-invasive ventilation, *SAPS* Simplified Acute Physiology Score

The comparator in four trials was standard oxygen via nasal prongs or facemask [28–30, 34], non-invasive ventilation in two trials [27, 31], and both standard oxygen and non-invasive ventilation arms in one trial [26]. Oxygen dose, flow rate, and level of pressure support were variably reported. Therapeutic indications ranged from mild to severe hypoxemic respiratory failure. Indications for study therapy were heterogeneous and included pre-oxygenation for hypoxemic patients requiring emergent endotracheal intubation (duration = 4 min) [28], oxygen support during bronchoscopy in hypoxemic patients in the ICU (duration ~ 65 min) [31], and oxygen support during admission for hypoxemia in the emergency department [30, 34] or ICU [26, 27, 29, 35] (duration from 1 h up to resolution of hypoxemia or failure of therapy) (Table 1). Based on stated patient population, intervention arms, or study design, we excluded 2011 citations after title and abstract screening. We excluded two trials after full-text screening due to absence of outcomes of interest [35] and inappropriate study design (cross-over) [13]. While publication bias could not be statistically assessed due to the number of published trials, there were no completed but unpublished trials in the World Health Organization’s Clinical Trial Registry Platform.

### Primary outcome

**Fig. 3:**
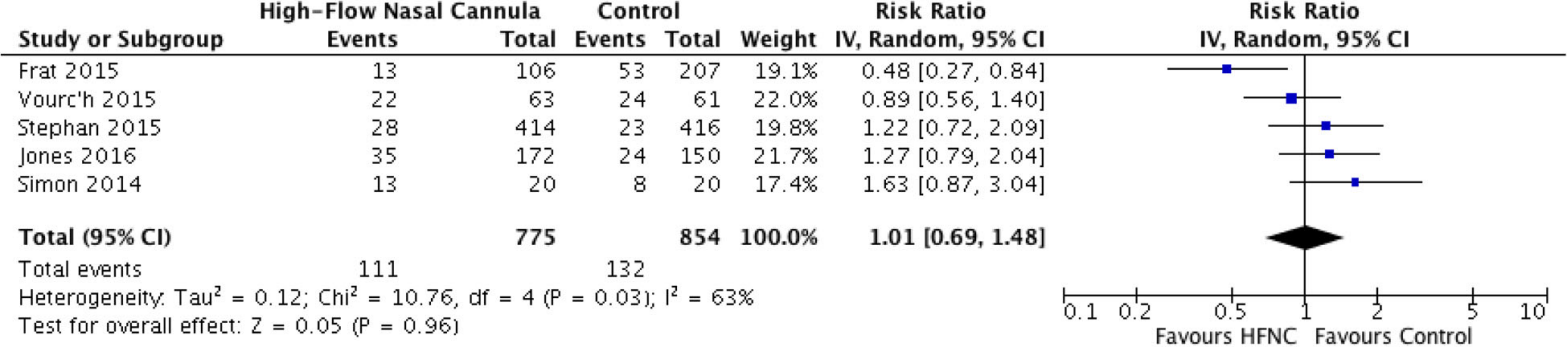
Mortality: high-flow oxygen via nasal cannulae vs. non-invasive ventilation or standard oxygen therapy. Boxes and horizontal lines represent point estimates and 95% confidence intervals, varying in size according to the weight in the analysis

High-flow oxygen via nasal cannula compared to standard oxygen or non-invasive ventilation was not associated with differential rates of mortality (RR 1.01 (95% CI, 0.69–1.48; *I* ^2^ = 63%; 1629 patients; five trials)) [26–28, 31, 34] (Fig. 3). We graded the overall strength of the evidence for a mortality effect as very low (Additional file 4).

#### Subgroup analysis

In patients randomized to receive oxygen support via high-flow nasal cannulae versus non-invasive ventilation, the risk ratio for death was 1.02 (95% CI, 0.53–1.96; *I* ^2^ = 69%; 1034 patients; 3 trials) [26, 27, 31]. In patients randomized to receive oxygen via high-flow nasal cannula versus standard oxygen therapy, the risk ratio for death was 0.94 (95% CI, 0.63–1.39; *I* ^2^ = 36%; 595 patients; 3 trials) [26, 28, 34] (Additional file 5). For other a priori specified subgroup analyses including study population, treatment location, and treatment protocol duration, high-flow oxygen was not associated with significant differences in mortality (Additional file 5). In a post hoc subgroup analysis, no significant mortality difference was found when excluding the single non-inferiority trial [27].

### Secondary outcomes

#### Dyspnea

Reported measures of dyspnea varied in scale and timing, limiting conclusive meta-analysis. In a trial of ICU patients with acute hypoxemia, high-flow oxygen via nasal cannulae was associated with significantly improved dyspnea after 1 h of therapy compared to non-invasive ventilation or standard oxygen (percent improvement 75.6% vs. 58.3% vs. 41.9% respectively, *p* < 0.001) [26]. In a trial of ED patients with acute hypoxemia, high-flow oxygen via nasal cannulae was also associated with significantly improved dyspnea at 1 h as compared to standard oxygen therapy (dyspnea score mean ± SD, 2.0 ± 1.8 high-flow nasal cannulae vs. 3.8 ± 2.3 standard oxygen; *p* = 0.01) [30]. In a trial of immunosuppressed ICU patients with hypoxemic respiratory failure, a significant difference in dyspnea grade at 120 min was not observed (median 3, IQR 2 to 6, high-flow nasal cannulae vs. 3, 1 to 6, standard oxygen) [29]. In a non-inferiority trial of post-cardiac surgical ICU patients with acute hypoxemic respiratory failure, there were no significant differences in dyspnea scores for patients receiving high-flow oxygen via nasal cannulae vs. non-invasive ventilation on serial assessments over the first 3 days of therapy [27].

#### Patient tolerance

High-flow oxygen via nasal cannulae were associated with significantly improved self-reported patient comfort after 60 min of therapy in two trials of ICU and ED patients (SMD − 0.63, 95% CI − 1.11 to − 0.15, *I* ^2^ = 54%, 126 patients, 2 trials) [26, 30]. Not all trials, however, reported enhanced patient tolerability of high-flow oxygen via nasal cannulae. In a trial of immunosuppressed ICU patients, a significant difference in patient-reported discomfort after 120 min of therapy was not observed (median, IQR 3, 1–5 high-flow nasal cannulae vs. 3, 0–5 standard oxygen) [29]. Likewise, a non-inferiority trial of cardiac surgical ICU patients with hypoxemic respiratory failure found no significant differences in patient-reported tolerability with high-flow oxygen via nasal cannulae compared to non-invasive ventilation over the first 3 days of therapy [27].

#### Physiologic variables

The pooled PaO_2_:FiO_2_ ratio at 6 to 12 h was significantly *lower* in two trials of ICU patients receiving oxygen via high-flow nasal cannulae compared to non-invasive ventilation or standard oxygen for hypoxemic respiratory failure (MD − 53.34, 95% CI − 71.95 to − 34.72, *I* ^2^ = 61%, 1143 patients) [26, 27]. The baseline PaO_2_:FiO_2_ ratios were similar in both groups. Similarly, in a trial of ICU patients with hypoxemic respiratory failure undergoing bronchoscopy, a lower mean PaO_2_:FiO_2_ ratio in patients randomized to high-flow oxygen via nasal cannulae compared to non-invasive ventilation in the immediate pre- and 50-min post-bronchoscopy period was observed [31]; however, this difference was non-significant at 24 h post-bronchoscopy [31]. Pooled mean differences in pH and PaCO_2_ were non-significant after 6 to 12 h of study therapy in two published trials [26, 27].

### Safety outcomes

High-flow oxygen via nasal cannulae was not associated with differential rates of endotracheal intubation and mechanical ventilation when compared to non-invasive ventilation or standard oxygen therapy (risk ratio, 0.85; 95% CI, 0.70–1.04; *I* ^2^ = 0%, 1605 patients; 5 trials) [26, 27, 29, 31, 34]. High-flow oxygen via nasal cannulae was not associated with statistically significant differential rates of cardio-respiratory arrest compared to non-invasive ventilation or standard oxygen therapy (risk ratio, 0.65; 95% CI, 0.26–1.62; *I*2 = 0%, 759 patients; 3 trials) [26, 28, 34]. The incidence, severity and duration of delirium and skin breakdown were infrequently reported (Table 2).

**Table 2:** Secondary and safety outcome measures

Outcome or subgroup		Participants		Effect estimate (95% CI)	*I* ^2^ (UCI)
	Studies	Intervention	Control		
Patient tolerance [26, 30]	2	*n* = 126	*n* = 227	SMD − 0.63 (− 1.11, − 0.15)	54%
Intubation [26, 27, 29, 31, 34]	5	113/764	176/841	RR 0.85 (0.70, 1.04)	0% (0%, 74%)
Cardio-respiratory arrest [26–28, 34]	3	5/341	13/207	RR 0.65 (0.26, 1.62)	0% (0%, 87%)
PaO_2_:FiO_2_^a^ [26, 27]	2	*n* = 520	*n* = 623	MD − 53.34 (− 71.95, − 34.72)	61%
pH^a^ [26, 27]	2	*n* = 520	*n* = 623	MD 0.01 (0.00, 0.01)	36%
PaCO_2_^a^ [26, 27]	2	*n* = 520	*n* = 623	MD − 0.66 (− 1.47, 0.16)	67%

*HFNC* high-flow nasal cannulae, *I* ^*2*^ I-squared, *UCI* uncertainty intervals

^a^Rating at 6–12 h post-randomization

### Trial sequential analysis

Trial sequential analysis was performed for mortality based on a relative risk reduction (RRR) of 0.26, a type I error of 0.05 and a type II error of 0.8. Using a random-effects model, accounting for heterogeneity (*I* ^*2*^ = 64%*)* in our sample, the required information size for the outcome of mortality was not reached (*n* = 6428). The boundaries for benefit, harm, or futility were not reached (Additional file 6)**.**

## Discussion

In our systematic review of critically ill patients with hypoxemic respiratory failure, oxygen therapy via high-flow nasal cannulae compared with non-invasive ventilation or standard oxygen was not associated with a significant difference in mortality. The effects on patient-reported dyspnea and patient tolerability were inconsistent. High-flow oxygen via nasal cannulae may be associated with reduced PaO_2_:FiO_2_ ratios. Compared to non-invasive ventilation or standard oxygen therapy, we found no difference in the rates of endotracheal intubation and cardio-respiratory arrest in patients receiving high-flow oxygen via nasal cannulae.

The absence of a mortality difference in patients receiving high-flow oxygen via nasal cannulae may reflect the inclusion of patients with mild to severe hypoxemic respiratory failure across a range of severity of illness scores; both within and between included trials. The dose and duration of study interventions were similarly variable with two trials applying the intervention for less than 2 h [28, 31]. The pooled estimate for mortality, however, remained non-significant when the effect of these short duration interventions was removed during subgroup analysis. The strength of evidence for our primary mortality outcome was classified as very low using the GRADE framework due to the inclusion of small pilot trials with a lack of biological plausibility of a mortality effect from such short duration interventions [28, 31] (range 4–120 min). None of the included trials were individually designed or powered to detect mortality as a primary outcome. A post hoc trial sequential analysis revealed that the required information size for the primary outcome mortality was not reached, suggesting that ongoing equipoise exists regarding a differential effect of HFNC on mortality.

While dyspnea and patient tolerability reflect clinically important patient-centered secondary outcomes, heterogeneity in the measurement and reporting or these outcomes precluded meta-analysis. Standardized, validated scales of dyspnea and comfort would, however, facilitate comparative evaluations and knowledge synthesis of future research. Detecting an association between high-flow oxygen via nasal cannulae and *lower* PaO_2_:FiO_2_ ratios was unanticipated. In the individual trials from which PaO_2_:FiO_2_ outcomes were abstracted high-flow nasal cannulae were associated with either a mortality benefit [26] or non-inferiority [27]. While commonly used to characterize the severity of ARDS [36], the ARDSnet investigators found that reductions in the PaO_2_:FiO_2_ ratio were inversely associated with mortality [37], and so, the predictive utility of this commonly reported surrogate outcome is questioned. Further, in the trials reporting PaO_2_:FiO_2_ ratio, FiO_2_ was estimated based on device settings in the majority of patients but calculated based on oxygen flow rate in others. Predictive methods of FiO_2_ estimation may be insensitive to variation in actual FiO_2_ delivered based on device used, nasal prong or mask interface and patient technique.

Due to rare events and under-reporting, differential safety outcomes may not have been fully captured by the included trials. While the pooled effect for high-flow oxygen on the incidence of cardio-respiratory arrest, skin breakdown and delirium failed to reach statistical significance, these clinically relevant outcomes were sparsely reported. Given that both non-invasive ventilation and high-flow oxygen via nasal cannulae have the potential to mask an underlying deterioration in oxygenation until a patient is in extremis, robust reporting of potential adverse clinical events is essential to evaluate efficacy in the context of potential harm [38, 39].

Although not part of our a priori established systematic review protocol, we observed that no trials presented cost-effectiveness or cost-utility analyses of high-flow oxygen. In one trial that reported a resource intensity variable (number of nursing interventions related to the study intervention) [27], high-flow oxygen was not associated with a differential nursing workload compared to non-invasive ventilation. When considering the role of high-flow nasal cannulae compared to other therapies, an economic evaluation would contribute valuable information to clinicians, healthcare resource managers, and funding agencies as the incremental cost-effectiveness for the provision of standard oxygen, non-invasive ventilation, and high-flow nasal cannulae would be expected to vary.

Our systemic review builds on and is an important refinement of four recently published systematic reviews evaluating high-flow oxygen therapy in heterogeneous groups of patients with respiratory failure or *at risk* for respiratory failure [40–43]. In contrast, our review included only trials where high-flow oxygen was used to treat established hypoxemic respiratory failure in critical illness. This distinction is important as the potential effect of high-flow oxygen therapy via nasal cannulae to prevent intubation in those at risk of respiratory failure may be entirely different from the potential effect of high-flow oxygen therapy as a treatment modality for patients with established hypoxemic respiratory failure. We restricted our sample to the inclusion of parallel group intervention trials whereas previously published reviews also included cross-over trials with added potential bias from carry-over effects. While none of the published reviews found a mortality benefit of high-flow oxygen via nasal cannulae, two found that high-flow oxygen via nasal cannulae was associated with lower intubation rates than conventional oxygen therapy [42, 43] while another found no difference in intubation rates [41]. This heterogeneity in summary effect measures highlights the sensitivity of meta-analysis to variable inclusion criteria and further supports the importance of precisely matching the clinical question with inclusion/exclusion criteria and study design. Our analysis further includes an evaluation of patient-oriented outcomes and clinically important safety variables.

Strengths of our systematic review and meta-analysis include the formulation of a focused question pertaining to a novel technology increasing in use; targeting clinically important efficacy and safety outcomes to inform best practice; implementation of a comprehensive, peer-reviewed search strategy with no language restriction; and appraisal of internal validity as well as the strength of the evidence using the Cochrane Risk of Bias Tool and GRADE methodology. As all meta-analyzed data were extracted from trials published within the last 3 years, the relevance and generalizability of our findings to current practice is high. We used extensive subgroup and sensitivity analyses to explore sources and the impact of variable treatment indications and duration. We also performed a trial sequential analysis that supported that a conclusion of no differential mortality effect between high-flow oxygen via nasal cannulae, and comparators may represent a type II error. Limitations of our study include low numbers of included trials, a high proportion of included trials at high risk of bias due to the lack of blinding, residual clinical heterogeneity relating to the indication for oxygen support, the duration of the interventions, and duration of follow-up. Small sample sizes of included trials, under powered for important outcomes is another limitation that we addressed through the use of trial sequential analysis. Incomplete reporting of safety measures may also have limited our ability to fully evaluate the safety of high-flow oxygen via nasal cannulae. There were too few published studies to statistically evaluate the presence or impact of publication bias.

## Conclusions

In critically ill adults with acute hypoxemic respiratory failure, high-flow oxygen via nasal cannula was not shown to be associated with significant differences in mortality, endotracheal intubation, or cardio-respiratory arrest compared to non-invasive ventilation or standard oxygen therapy. These conclusions should be interpreted in the context of residual heterogeneity between patient populations and the application of the high-flow oxygen among included trials. Potential benefits, including dyspnea and tolerance, in addition to safety outcomes and economic considerations require further evaluation.

## Additional files

Additional file 1: Study protocol. (DOCX 44 kb)

Additional file 2: Systematic review team members. (DOCX 10 kb)

Additional file 3: Ovid MEDLINE search strategy. (DOCX 14 kb)

Additional file 4: GRADE summary of evidence table. (DOCX 13 kb)

Additional file 5: Mortality: subgroup analysis. (DOCX 4447 kb)

Additional file 6: Trial sequential analysis for mortality. (DOCX 257 kb)

## Abbreviations

ED: Emergency department
FiO_2_: Fraction of inspired oxygen
GRADE: Grading of Recommendations Assessment, Development and Evaluation
HFNC: High-flow oxygen via nasal cannulae
ICU: Intensive care unit
IQR: Inter-quartile range
L: Liters
MD: Mean difference
NIV: Non-invasive ventilation
PaCO_2_: Partial pressure of arterial carbon dioxide
PaO_2_: Partial pressure of arterial oxygen
RR: Risk ratio
RRR: Relative risk reduction
SAPS: Simplified acute physiology score
SD: Standard deviation
SMD: Standardized mean difference

## References

[1] Papazian L, Corley A, Hess D (2016). Use of high-flow nasal cannula oxygenation in ICU adults: a narrative review. Intensive Care Med.

[2] Ram FS, Picot J, Lightowler J, Wedzicha JA (2004). Non-invasive positive pressure ventilation for treatment of respiratory failure due to exacerbations of chronic obstructive pulmonary disease. Cochrane Database Syst Rev.

[3] Vital FM, Ladeira MT, Atallah AN (2013). Non-invasive positive pressure ventilation (CPAP or bilevel NPPV) for cardiogenic pulmonary oedema. Cochrane Database Syst Rev.

[4] Burns KE, Meade MO, Premji A, Adhikari NK (2013). Noninvasive positive-pressure ventilation as a weaning strategy for intubated adults with respiratory failure. Cochrane Database Syst Rev.

[5] Keenan SP, Sinuff T, Cook DJ, Hill NS (2004). Does noninvasive positive pressure ventilation improve outcome in acute hypoxemic respiratory failure? A systematic review. Crit Care Med.

[6] Esteban A, Frutos-Vivar F, Muriel A (2013). Evolution of mortality over time in patients receiving mechanical ventilation. Am J Respir Crit Care Med.

[7] Thille AW, Contou D, Fragnoli C, Cordoba-Izquierdo A, Boissier F, Brun-Buisson C (2013). Non-invasive ventilation for acute hypoxemic respiratory failure: intubation rate and risk factors. Crit Care.

[8] Chanques G, Riboulet F, Molinari N (2013). Comparison of three high flow oxygen therapy delivery devices: a clinical physiological cross-over study. Minerva Anestesiol.

[9] Parke RL, Eccleston ML, McGuinness SP (2011). The effects of flow on airway pressure during nasal high-flow oxygen therapy. Respir Care.

[10] Sztrymf B, Messika J, Bertrand F (2011). Beneficial effects of humidified high flow nasal oxygen in critical care patients: a prospective pilot study. Intensive Care Med.

[11] Sztrymf B, Messika J, Mayot T, Lenglet H, Dreyfuss D, Ricard JD (2012). Impact of high-flow nasal cannula oxygen therapy on intensive care unit patients with acute respiratory failure: a prospective observational study. J Crit Care.

[12] Corley A, Caruana LR, Barnett AG, Tronstad O, Fraser JF (2011). Oxygen delivery through high-flow nasal cannulae increase end-expiratory lung volume and reduce respiratory rate in post-cardiac surgical patients. Br J Anaesth.

[13] Cuquemelle E, Pham T, Papon JF, Louis B, Danin PE, Brochard L (2012). Heated and humidified high-flow oxygen therapy reduces discomfort during hypoxemic respiratory failure. Respir Care.

[14] Roca O, Riera J, Torres F, Masclans JR (2010). High-flow oxygen therapy in acute respiratory failure. Respir Care.

[15] Frat JP, Brugiere B, Ragot S (2015). Sequential application of oxygen therapy via high-flow nasal cannula and noninvasive ventilation in acute respiratory failure: an observational pilot study. Respir Care.

[16] Kang BJ, Koh Y, Lim CM (2015). Failure of high-flow nasal cannula therapy may delay intubation and increase mortality. Intensive Care Med.

[17] Chandler J, Churchill R, Higgins J, Lasserson T, Tovey D (2013). Methodological standards for the conduct of new Cochrane Intervention Reviews.

[18] Liberati A, Altman DG, Tetzlaff J (2009). The PRISMA statement for reporting systematic reviews and meta-analyses of studies that evaluate health care interventions: explanation and elaboration. PLoS Med.

[19] Sampson M, McGowan J, Lefebvre C, Moher D, Grimshaw J (2008). PRESS: Peer Review of Electronic Search Strategies.

[20] Higgins JP, Altman DG, Gotzsche PC (2011). The Cochrane Collaboration’s tool for assessing risk of bias in randomised trials. BMJ.

[21] Higgins J, Green S (2011). Cochrane Handbook for Systematic Reviews of Interventions.

[22] Guyatt GH, Oxman AD, Vist GE (2008). GRADE: an emerging consensus on rating quality of evidence and strength of recommendations. BMJ.

[23] Higgins JP, Thompson SG (2002). Quantifying heterogeneity in a meta-analysis. Stat Med.

[24] Brok J, Thorlund K, Wetterslev J, Gluud C (2009). Apparently conclusive meta-analyses may be inconclusive––trial sequential analysis adjustment of random error risk due to repetitive testing of accumulating data in apparently conclusive neonatal meta-analyses. Int J Epidemiol.

[25] Wetterslev J, Jakobsen JC, Gluud C (2017). Trial Sequential Analysis in systematic reviews with meta-analysis. BMC Med Res Methodol.

[26] Frat JP, Thille AW, Mercat A (2015). High-flow oxygen through nasal cannula in acute hypoxemic respiratory failure. N Engl J Med.

[27] Stephan F, Barrucand B, Petit P (2015). High-flow nasal oxygen vs noninvasive positive airway pressure in hypoxemic patients after cardiothoracic surgery: a randomized clinical trial. JAMA.

[28] Vourc'h M, Asfar P, Volteau C (2015). High-flow nasal cannula oxygen during endotracheal intubation in hypoxemic patients: a randomized controlled clinical trial. Intensive Care Med.

[29] Lemiale V, Mokart D, Mayaux J (2015). The effects of a 2-h trial of high-flow oxygen by nasal cannula versus Venturi mask in immunocompromised patients with hypoxemic acute respiratory failure: a multicenter randomized trial. Critical Care (London, England).

[30] Rittayamai N, Tscheikuna J, Praphruetkit N, Kijpinyochai S (2015). Use of high-flow nasal cannula for acute dyspnea and hypoxemia in the emergency department. Respir Care.

[31] Simon M, Braune S, Frings D, Wiontzek AK, Klose H, Kluge S (2014). High-flow nasal cannula oxygen versus non-invasive ventilation in patients with acute hypoxaemic respiratory failure undergoing flexible bronchoscopy––a prospective randomised trial. Critical Care (London, England).

[32] Stephan F, Barrucand B, Petit P (2014). Bilevel positive airway pressure versus optiflow in hypoxemic patients after cardiothoracic surgery (the bipop study): a multicenter, randomized, noninferiority, open trial. American Journal of Respiratory and Critical Care Medicine Conference: American Thoracic Society International Conference, ATS.

[33] Frat JP, Thille A, Girault C, Ragot S (2013). FLORALI study (High-Flow Oxygen Therapy for the Resuscitation of Acute Lung Injury): use of nasal high-flow oxygen therapy in non-hypercapnic acute respiratory failure. Introduction to the study protocol. French. Reanimation.

[34] Jones PG, Kamona S, Doran O, Sawtell F, Wilsher M (2016). Randomized controlled trial of humidified high-flow nasal oxygen for acute respiratory distress in the emergency department: the HOT-ER study. Respir Care.

[35] Parke RL, McGuinness SP, Eccleston ML (2011). A preliminary randomized controlled trial to assess effectiveness of nasal high-flow oxygen in intensive care patients. Respir Care.

[36] Ferguson ND, Fan E, Camporota L (2012). The Berlin definition of ARDS: an expanded rationale, justification, and supplementary material. Intensive Care Med.

[37] Network A. Ventilation with lower tidal volumes as compared with traditional tidal volumes for acute lung injury and the acute respiratory distress syndrome. The Acute Respiratory Distress Syndrome Network. N Engl J Med 2000;342(18):1301-1308. 10.1056/NEJM200005043421801 10793162

[38] Hill NS (2000). Complications of noninvasive ventilation. Respir Care.

[39] Antón A, Güell R, Gómez J (2000). Predicting the result of noninvasive ventilation in severe acute exacerbations of patients with chronic airflow limitation. Chest.

[40] Nedel WL, Deutschendorf C, Moraes Rodrigues Filho E (2017). High-flow nasal cannula in critically ill subjects with or at risk for respiratory failure: a systematic review and meta-analysis. Respir Care.

[41] Monro-Somerville T, Sim M, Ruddy J, Vilas M, Gillies MA (2016). The effect of high-flow nasal cannula oxygen therapy on mortality and intubation rate in acute respiratory failure: a systematic review and meta-analysis. Crit Care Med.

[42] Ni YN, Luo J, Yu H (2017). Can high-flow nasal cannula reduce the rate of endotracheal intubation in adult patients with acute respiratory failure compared with conventional oxygen therapy and noninvasive positive pressure ventilation?: a systematic review and meta-analysis. Chest.

[43] Ou X, Hua Y, Liu J, Gong C, Zhao W (2017). Effect of high-flow nasal cannula oxygen therapy in adults with acute hypoxemic respiratory failure: a meta-analysis of randomized controlled trials. CMAJ.

