# Supplementary material for: High-flow oxygen via nasal cannulae in patients with acute hypoxemic respiratory failure: a systematic review and meta-analysis
Source: Syst Rev. 2017 Oct 16;6:202. doi: 10.1186/s13643-017-0593-5 (PMC5644261; doi:10.1186/s13643-017-0593-5)
Supplement: Supplementary file 1 — Study protocol. (DOCX 44 kb) [file 13643_2017_593_MOESM1_ESM.docx]

Appendix 3 – Study Protocol

High-flow oxygen via nasal cannulae in patients with acute hypoxemic respiratory failure:

A systematic review and meta-analysis protocol

Review Authors: Murdoch Leeies^1,2,3^, Eric Flynn^1^, Alexis F Turgeon^4^, Bojan Paunovic^2^, Hal Loewen^5^, Rasheda Rabbani^3,6^, Ahmed M. Abou-Setta^3,6^, Niall D. Ferguson^7^, Ryan Zarychanski^2,3,6^

Affiliations:

^1^ Department of Emergency Medicine, University of Manitoba, Winnipeg, Manitoba, Canada

^2^ Department of Internal Medicine, Section of Critical Care, University of Manitoba, Winnipeg, Manitoba, Canada

^3^ Department of Community Health Sciences, University of Manitoba, Winnipeg, Manitoba, Canada

^4^ Division of Critical Care Medicine, Department of Anesthesiology, Université Laval, Québec City, Québec, Canada

^5^ Neil John Mclean Library, University of Manitoba, Winnipeg, Manitoba, Canada

^6^ George & Fay Yee Center for Healthcare Innovation, University of Manitoba/Winnipeg Regional Health Authority, Winnipeg, Manitoba, Canada

^7^ Department of Medicine, Division of Respirology, University Health Network and Mount Sinai Hospital; Toronto General Research Institute; Interdepartmental Division of Critical Care Medicine, Departments of Medicine and Physiology, and Institute of Health Policy, Management and Evaluation, University of Toronto, Toronto, Ontario, Canada

# Background

Acute hypoxemic respiratory failure is widely prevalent in acutely ill patients. Supplemental oxygen therapy is administered via nasal prongs, facemasks, non-invasive or invasive ventilation modalities to correct hypoxemia. Standard nasal prong or facemask systems are limited to a maximum delivery of 15-20 L/min of oxygen. Traditional non-invasive positive pressure ventilation has been applied with benefit to heterogeneous populations with acute respiratory failure including those with chronic obstructive pulmonary disease[^2^](#_ENREF_2), cardiogenic pulmonary edema[^3^](#_ENREF_3) and as a weaning strategy in adults intubated for acute respiratory failure[^4^](#_ENREF_4). In a systematic review of non-invasive positive pressure ventilation in patients with acute hypoxemic respiratory failure, however, there was significant heterogeneity and the effect on mortality was unclear[^5^](#_ENREF_5). The need for invasive ventilation in patients with acute hypoxemic respiratory failure is associated with significant morbidity and mortality[^6^](#_ENREF_6)^,^[^7^](#_ENREF_7).

High flow oxygen via nasal cannula is a non-invasive therapy where heated, humidified oxygen is delivered via large-bore nasal cannula at flow rates up to 60 L/min. The fraction of inspired oxygen can be titrated to 100% and the mean airway pressure increases with increasing flow[^8^](#_ENREF_8)^,^[^9^](#_ENREF_9). Observational studies support that high flow oxygen via nasal cannula may be associated with improved oxygenation, decreased respiratory rate, increased lung volumes and improved patient comfort as compared to standard oxygen therapy[^10-14^](#_ENREF_10) and be better tolerated than non-invasive ventilation[^15^](#_ENREF_15). The efficacy and safety of high flow oxygen via nasal cannula for acute hypoxemic respiratory failure in randomized trials, however, remains uncertain.

The purpose of this systematic review is to identify, critically appraise and meta-analyze data from prospective randomized trials comparing high-flow oxygen via nasal cannula in adult patients with acute hypoxemic respiratory failure with other interventions.

## Free form Question:

In adult patients with acute hypoxemic respiratory failure what is the comparative efficacy of high-flow oxygen via nasal cannula compared with other respiratory therapies with respect to mortality, incidence of intubation, patient tolerability and other patient-centred outcomes?

## Structured Question:

*Population*

- Adults with acute hypoxemic respiratory failure;

*Intervention*

- High-flow, humidified oxygen via nasal cannula;

*Comparator*

- Any comparator including standard care, active comparator, or dummy treatment;

*Outcomes*

- Primary Outcome:
  - Incidence of mortality at longest duration of follow-up;
- Secondary Outcomes:
  - Patient-reported tolerability;
  - Patient-reported dyspnea;
  - PaO_2_:FiO_2_;
  - PaCO_2_;
  - pH;
- Safety Outcomes:
  - Incidence of endotracheal intubation;
  - Delirium (incidence, severity, duration);
  - Cardio-respiratory arrest;
  - Skin breakdown.

*Study design*

- Prospective, randomized, controlled trials.

### Eligibility Criteria

*Inclusion Criteria:*

1. Adult (>17 years old) patients with author-defined acute hypoxemic respiratory failure (80% of population);
2. Prospective, randomized, controlled trials;
3. High-flow, humidified oxygen via nasal cannula administration.

*Exclusion Criteria:*

1. Animal studies;
2. Observational and/or quasi-experimental methods (i.e. cohort or case-control studies, alternate randomization schemes, etc.);
3. Prophylactic application of high-flow oxygen.

# Systematic Review Team Members

One clinician investigator trainee (ML) with emergency medicine and critical care specialization will coordinate all facets of the review, including development of the literature search, screening relevant materials, extracting and analyzing data, and manuscript preparation. A second researcher will assist throughout the review process by screening relevant material, extracting data and assessing trial risk of bias in duplicate (EF); One intensivist clinician scientist will provide direct supervision, content expertise and methodological input in addition to resolution of disagreement among reviewers (RZ). Two academic librarians will contribute to the development (HL) and subsequent peer-review (BS) of the search strategy. Three additional critical care physicians with subspecialties in anesthesiology, internal medicine and respirology (AT, BP, NF) and one knowledge synthesis expert (AMAS) with experience conducting systematic reviews will provide content expertise and methodological advice; one senior statistician and methodologist (RR) with extensive systematic review experience will provide statistical expertise and oversight.

# Search strategy for identification of studies

We will search CENTRAL (the Cochrane Library – Wiley), EMBASE (Ovid), and PubMed/ Medline (National Library of Medicine) from inception to present using individualized search strategies prepared for each database. We will perform a forward search in Scopus and Web of Science to identify additional relevant citations. Finally, in order to identify ongoing or planned trials we will search the World Health Organization’s International Clinical Trials Registry Platform. The search strategy for MEDLINE is presented in Appendix 1.

In addition to electronic searching, we will search abstracts and conference proceedings for the following societies (2012 – 2016): *American College of Emergency Physicians, American Thoracic Society, Canadian Association of Emergency Physicians, Canadian Critical Care Society, European Society of Intensive Care Medicine, and Society of Critical Care Medicine*. The reference lists of relevant narrative and systematic reviews as well as all included trials will be hand-searched for possible relevant citations. Reference Management will be performed using EndNote™ (ver. X7.5). We will create a PRIMSA flow diagram illustrating the number of records and full-text reports reviewed and inclusion or exclusion.

We will employ a multi-step process for study selection. Initially, two reviewers will independently screen the titles and abstracts of search results to determine if each study meets inclusion criteria. Each report will be classified as: *include*, *exclude*, *unclear*, or *duplicate of another citation*. All conflicts will be resolved by consensus between the two primary reviewers or by third-party adjudication. The full text of all reports classified as *include* will be retrieved for formal review. Next, the two reviewers will independently assess the full text of each report, employing a standardized data extraction form that outlines the predetermined inclusion and exclusion criteria. The tool will be pilot tested on a sample of studies. After the data extraction tool is tested, disagreements will be resolved by consensus between the two primary reviewers or by third-party adjudication, if required.

# Data abstraction and management

Data will be abstracted using a standardized form and entered into a Microsoft Excel™ database (Microsoft Corp., Redmond, WA). The data extraction tool will be pilot tested on a sample of studies. Two reviewers will independently extract data from individual studies, with disagreements resolved through consensus or third-party adjudication if consensus cannot be achieved. The following data will be extracted from each study: author identification, year of publication, language of publication, source of study funding, study design, methodological quality criteria (see below), study population, patient characteristics (age, sex, reason for acute hypoxemic respiratory failure, SAPS II score or other severity of illness score, arterial blood gas results, intervention (device utilized, fraction of inspired oxygen) and its comparator, and results reported for the outcomes of interest.

# Risk of Bias Assessment

We will assess the internal validity of included trials using the Cochrane Collaboration Risk of Bias tool [^20^](#_ENREF_20)^,^[^21^](#_ENREF_21). This tool includes six domains (sequence generation, allocation concealment, blinding, incomplete outcome data, selective outcome reporting, and “other” sources of bias) and additionally classifies the overall risk of bias. If one or more individual domains are assessed as having a high risk of bias, the overall score will be rated as having a high risk of bias. All domains must be rated as having a low risk of bias for the overall risk of bias to be classified as low. In cases of unclear risk of bias or mixed assessments of low and unclear risk of bias, the overall score will be classified as having an unclear risk of bias. Information regarding methodological quality will be used to guide sensitivity analyses and explore sources of heterogeneity.

# Measures of Treatment Effect

We will analyze data from included studies using RevMan (version 5.3.5). If the data are sufficiently statistically and clinically homogeneous we will conduct a formal meta-analysis. Pooled continuous data will be expressed as mean differences, or standardized mean differences where multiple scales are used to measure the same outcome, with 95% confidence intervals. Pooled dichotomous data will be presented as risk ratios, or for rare outcomes using Peto-Odds Ratios. For the primary outcome (e.g. mortality), we will report the absolute risk. We will explore and quantify statistical heterogeneity of the data using the I-squared test[^23^](#_ENREF_23). Sub-group and sensitivity analyses will be conducted if significant heterogeneity is suspected. Publication bias will be assessed via funnel plot methods as appropriate[^44^](#_ENREF_44).

# Subgroup / Sensitivity Analysis

The following *a priori* subgroup and sensitivity analyses are proposed. Such analyses will depend on the number of studies included and the availability of appropriate outcomes and covariates.

*Methodological*

Sponsor (Industry Funded vs. non-industry funded)

Risk of bias (Low risk of bias vs. unclear/ high risk of bias)

Source (Published vs. Grey literature/ abstracts/ conf. proceedings)

*Clinical*

Type of comparator (non-invasive ventilation vs. standard oxygen)

Patient population (emergency department vs. intensive care unit vs.

cardiac surgical)

Dose (fractional inspired oxygen, cut offs to be determined)

Duration of study protocol (as per included studies)

# Conflict of Interest Statement

None of the authors has any conflicts of interest that could affect the design or analysis of this systematic review.

Protocol References

1. Papazian L, Corley A, Hess D, et al. Use of high-flow nasal cannula oxygenation in ICU adults: a narrative review. *Intensive Care Med.* 2016.

2. Ram FS, Picot J, Lightowler J, Wedzicha JA. Non-invasive positive pressure ventilation for treatment of respiratory failure due to exacerbations of chronic obstructive pulmonary disease. *Cochrane Database Syst Rev.* 2004(3):CD004104.

3. Vital FM, Ladeira MT, Atallah AN. Non-invasive positive pressure ventilation (CPAP or bilevel NPPV) for cardiogenic pulmonary oedema. *Cochrane Database Syst Rev.* 2013;5:CD005351.

4. Burns KE, Meade MO, Premji A, Adhikari NK. Noninvasive positive-pressure ventilation as a weaning strategy for intubated adults with respiratory failure. *Cochrane Database Syst Rev.* 2013;12:CD004127.

5. Keenan SP, Sinuff T, Cook DJ, Hill NS. Does noninvasive positive pressure ventilation improve outcome in acute hypoxemic respiratory failure? A systematic review. *Critical Care Medicine.* 2004;32(12):2516-2523.

6. Esteban A, Frutos-Vivar F, Muriel A, et al. Evolution of mortality over time in patients receiving mechanical ventilation. *Am J Respir Crit Care Med.* 2013;188(2):220-230.

7. Thille AW, Contou D, Fragnoli C, Cordoba-Izquierdo A, Boissier F, Brun-Buisson C. Non-invasive ventilation for acute hypoxemic respiratory failure: intubation rate and risk factors. *Crit Care.* 2013;17(6):R269.

8. Chanques G, Riboulet F, Molinari N, et al. Comparison of three high flow oxygen therapy delivery devices: a clinical physiological cross-over study. *Minerva Anestesiol.* 2013;79(12):1344-1355.

9. Parke RL, Eccleston ML, McGuinness SP. The effects of flow on airway pressure during nasal high-flow oxygen therapy. *Respir Care.* 2011;56(8):1151-1155.

10. Sztrymf B, Messika J, Bertrand F, et al. Beneficial effects of humidified high flow nasal oxygen in critical care patients: a prospective pilot study. *Intensive Care Med.* 2011;37(11):1780-1786.

11. Sztrymf B, Messika J, Mayot T, Lenglet H, Dreyfuss D, Ricard JD. Impact of high-flow nasal cannula oxygen therapy on intensive care unit patients with acute respiratory failure: a prospective observational study. *J Crit Care.* 2012;27(3):324 e329-313.

12. Corley A, Caruana LR, Barnett AG, Tronstad O, Fraser JF. Oxygen delivery through high-flow nasal cannulae increase end-expiratory lung volume and reduce respiratory rate in post-cardiac surgical patients. *Br J Anaesth.* 2011;107(6):998-1004.

13. Cuquemelle E, Pham T, Papon JF, Louis B, Danin PE, Brochard L. Heated and humidified high-flow oxygen therapy reduces discomfort during hypoxemic respiratory failure. *Respir Care.* 2012;57(10):1571-1577.

14. Roca O, Riera J, Torres F, Masclans JR. High-flow oxygen therapy in acute respiratory failure. *Respir Care.* 2010;55(4):408-413.

15. Frat JP, Brugiere B, Ragot S, et al. Sequential application of oxygen therapy via high-flow nasal cannula and noninvasive ventilation in acute respiratory failure: an observational pilot study. *Respir Care.* 2015;60(2):170-178.

16. Kang BJ, Koh Y, Lim CM, et al. Failure of high-flow nasal cannula therapy may delay intubation and increase mortality. *Intensive Care Medicine.* 2015;41(4):623-632.

17. Chandler J, Churchill R, Higgins J, Lasserson T, Tovey D. *Methodological standards for the conduct of new Cochrane Intervention Reviews.* Vol 2.3: The Cochrane Library; 2013.

18. Liberati A, Altman DG, Tetzlaff J, et al. The PRISMA statement for reporting systematic reviews and meta-analyses of studies that evaluate health care interventions: explanation and elaboration. *PLoS Med.* 2009;6(7):e1000100.

19. Sampson M, McGowan J, Lefebvre C, Moher D, Grimshaw J. *PRESS: Peer Review of Electronic Search Strategies.* Canadian Agency for Drugs and Technologies in Health;2008.

20. Higgins JP, Altman DG, Gotzsche PC, et al. The Cochrane Collaboration's tool for assessing risk of bias in randomised trials. *BMJ.* 2011;343:d5928.

21. Cochrane Handbook for Systematic Reviews of Interventions. In: Higgins J, Green S, eds: The Cochrane Collaboration; 2011: [http://www.cochrane-handbook.org/](http://www.cochrane-handbook.org).

22. Guyatt GH, Oxman AD, Vist GE, et al. GRADE: an emerging consensus on rating quality of evidence and strength of recommendations. *BMJ.* 2008;336(7650):924-926.

23. Higgins JP, Thompson SG. Quantifying heterogeneity in a meta-analysis. *Stat Med.* 2002;21(11):1539-1558.

24. Brok J, Thorlund K, Wetterslev J, Gluud C. Apparently conclusive meta-analyses may be inconclusive--Trial sequential analysis adjustment of random error risk due to repetitive testing of accumulating data in apparently conclusive neonatal meta-analyses. *Int J Epidemiol.* 2009;38(1):287-298.

25. Wetterslev J, Jakobsen JC, Gluud C. Trial Sequential Analysis in systematic reviews with meta-analysis. *BMC Med Res Methodol.* 2017;17(1):39.

26. Frat JP, Thille AW, Mercat A, et al. High-flow oxygen through nasal cannula in acute hypoxemic respiratory failure. *New England Journal of Medicine.* 2015;372(23):2185-2196.

27. Stephan F, Barrucand B, Petit P, et al. High-Flow Nasal Oxygen vs Noninvasive Positive Airway Pressure in Hypoxemic Patients After Cardiothoracic Surgery: A Randomized Clinical Trial. *Jama.* 2015;313(23):2331-2339.

28. Vourc'h M, Asfar P, Volteau C, et al. High-flow nasal cannula oxygen during endotracheal intubation in hypoxemic patients: a randomized controlled clinical trial. *Intensive Care Medicine.* 2015;41(9):1538-1548.

29. Lemiale V, Mokart D, Mayaux J, et al. The effects of a 2-h trial of high-flow oxygen by nasal cannula versus Venturi mask in immunocompromised patients with hypoxemic acute respiratory failure: a multicenter randomized trial. *Critical Care (London, England).* 2015;19:380.

30. Rittayamai N, Tscheikuna J, Praphruetkit N, Kijpinyochai S. Use of High-Flow Nasal Cannula for Acute Dyspnea and Hypoxemia in the Emergency Department. *Respiratory Care.* 2015;60(10):1377-1382.

31. Simon M, Braune S, Frings D, Wiontzek AK, Klose H, Kluge S. High-flow nasal cannula oxygen versus non-invasive ventilation in patients with acute hypoxaemic respiratory failure undergoing flexible bronchoscopy--a prospective randomised trial. *Critical Care (London, England).* 2014;18(6):712.

32. Stephan F, Barrucand B, Petit P, et al. Bilevel positive airway pressure versus optiflow in hypoxemic patients after cardiothoracic surgery (the bipop study): A multicenter, randomized, noninferiority, open trial. *American Journal of Respiratory and Critical Care Medicine Conference: American Thoracic Society International Conference, ATS.* 2014;189(no pagination).

33. Frat JP, Thille A, Girault C, Ragot S. FLORALI study (High-Flow Oxygen Therapy for the Resuscitation of Acute Lung Injury): Use of nasal high-flow oxygen therapy in non-hypercapnic acute respiratory failure. Introduction to the study protocol. French. *Reanimation.* 2013;22(1):90-99.

34. Jones PG, Kamona S, Doran O, Sawtell F, Wilsher M. Randomized Controlled Trial of Humidified High-Flow Nasal Oxygen for Acute Respiratory Distress in the Emergency Department: The HOT-ER Study. *Respir Care.* 2016;61(3):291-299.

35. Parke RL, McGuinness SP, Eccleston ML. A preliminary randomized controlled trial to assess effectiveness of nasal high-flow oxygen in intensive care patients. *Respiratory Care.* 2011;56(3):265-270.

36. Ferguson ND, Fan E, Camporota L, et al. The Berlin definition of ARDS: an expanded rationale, justification, and supplementary material. *Intensive Care Med.* 2012;38(10):1573-1582.

37. Network A. Ventilation with lower tidal volumes as compared with traditional tidal volumes for acute lung injury and the acute respiratory distress syndrome. The Acute Respiratory Distress Syndrome Network. *N Engl J Med.* 2000;342(18):1301-1308.

38. Hill NS. Complications of nonivasive ventilation. *Respir Care.* 2000;45(5):480-481.

39. Antón A, Güell R, Gómez J, et al. Predicting the Result of Noninvasive Ventilation in Severe Acute Exacerbations of Patients With Chronic Airflow Limitation. *Chest.* 2000;117(3):828-833.

40. Nedel WL, Deutschendorf C, Moraes Rodrigues Filho E. High-Flow Nasal Cannula in Critically Ill Subjects With or at Risk for Respiratory Failure: A Systematic Review and Meta-Analysis. *Respir Care.* 2017;62(1):123-132.

41. Monro-Somerville T, Sim M, Ruddy J, Vilas M, Gillies MA. The Effect of High-Flow Nasal Cannula Oxygen Therapy on Mortality and Intubation Rate in Acute Respiratory Failure: A Systematic Review and Meta-Analysis. *Crit Care Med.* 2016.

42. Ni YN, Luo J, Yu H, et al. Can High-flow Nasal Cannula Reduce the Rate of Endotracheal Intubation in Adult Patients With Acute Respiratory Failure Compared With Conventional Oxygen Therapy and Noninvasive Positive Pressure Ventilation?: A Systematic Review and Meta-analysis. *Chest.* 2017;151(4):764-775.

43. Ou X, Hua Y, Liu J, Gong C, Zhao W. Effect of high-flow nasal cannula oxygen therapy in adults with acute hypoxemic respiratory failure: a meta-analysis of randomized controlled trials. *CMAJ.* 2017;189(7):E260-E267.

44. Ioannidis JP, Trikalinos TA. The appropriateness of asymmetry tests for publication bias in meta-analyses: a large survey. *CMAJ.* 2007;176(8):1091-1096.
