# Supplementary material for: High-flow oxygen via nasal cannulae in patients with acute hypoxemic respiratory failure: a systematic review and meta-analysis
Source: Syst Rev. 2017 Oct 16;6:202. doi: 10.1186/s13643-017-0593-5 (PMC5644261; doi:10.1186/s13643-017-0593-5)
Supplement: Supplementary file 2 — Systematic review team members. (DOCX 10 kb) [file 13643_2017_593_MOESM2_ESM.docx]

Appendix 1 – Systematic Review Team Members

One clinician investigator trainee (ML) with emergency medicine and critical care specialization coordinated all facets of the review, including development of the literature search, screening relevant materials, extracting and analyzing data, and manuscript preparation. A second researcher assisted throughout the review process by screening relevant material, extracting data and assessing trial risk of bias in duplicate (EF); one intensivist clinician scientist provided direct supervision, content expertise and methodological input in addition to resolution of disagreement among reviewers (RZ). Two academic librarians contributed to the development (HL) and subsequent peer-review (BS) of the search strategy. Three additional critical care physicians with subspecialties in anesthesiology, internal medicine and pulmonology (AT, BP, NF) and one knowledge synthesis expert (AMAS) with experience conducting systematic reviews provided content expertise and methodological advice; one senior statistician and methodologist (RR) with extensive systematic review experience provided statistical expertise and oversight.
