# Supplementary material for: High-flow oxygen via nasal cannulae in patients with acute hypoxemic respiratory failure: a systematic review and meta-analysis
Source: Syst Rev. 2017 Oct 16;6:202. doi: 10.1186/s13643-017-0593-5 (PMC5644261; doi:10.1186/s13643-017-0593-5)
Supplement: Supplementary file 3 — Ovid MEDLINE search strategy. (DOCX 14 kb) [file 13643_2017_593_MOESM3_ESM.docx]

Appendix 2 – Ovid MEDLINE Search Strategy

1. Anoxia/

2. Respiration Disorders/

3. Respiratory Aspiration/

4. Acute Chest Syndrome/

5. Respiratory Insufficiency/

6. Severe Acute Respiratory Syndrome/

7. Respiratory Distress Syndrome, Adult/

8. human ARDS.tw,kw.

9. hypoxem$.tw,kw.

10. hypoxidosis.tw,kw.

11. (hypoxia or hypoxic).tw,kw.

12. ((breathing or ventilat$ or respirat$ or oxygen) adj5 (insufficien$ or deficien$ or fail$ or distress$ or depress$)).tw,kw.

13. acute respiratory syndrome.tw,kw.

14. anoxia$.tw,kw.

15. anox?emi$.tw,kw.

16. or/1-15

17. Noninvasive Ventilation/

18. Optiflow.tw,kw.

19. ((High-flow or highflow or high flow or high frequency) adj5 (oxygen$ or O2)).tw,kw.

20. ((High-flow or highflow or high flow or high frequency) adj5 (cannula$ or prong$ or nasal or nose)).tw,kw.

21. ((oxygen$ or O2$) adj5 (cannula$ or prong$ or nasal or catheter$)).tw,kw.

22. (hfnc or hfnp or hhfnox).tw,kw.

23. (((bilevel or bi-level) and positive airway pressure$) or bipap).tw,kw.

24. ((non-invasive or non invasive or noninvasive) adj5 (respirat$ or ventilat$)).tw,kw.

25. or/17-24

26. 16 and 25

27. randomized controlled trial.pt.

28. controlled clinical trial.pt.

29. clinical trials as topic.sh.

30. (randomi#ed or randomly or RCT$1 or placebo*).tw.

31. ((singl* or doubl* or trebl* or tripl*) adj (mask* or blind* or dumm*)).tw.

32. trial.ti.

33. or/27-32

34. 26 and 33

35. exp Animals/ not (exp Animals/ and Humans/)

36. 34 not 35

37. Adolescent/ not (exp Adult/ and Adolescent/)

38. exp Child/ not (exp Adult/ and exp Child/)

39. exp Infant/ not (exp Adult/ and exp Infant/)

40. or/37-39

41. 36 not 40

42. letter.pt. not (letter.pt. and randomized controlled trial/)

43. (comment or editorial or interview or news).pt.

44. or/42-43

45. 41 not 44
