# Supplementary material for: High-flow oxygen via nasal cannulae in patients with acute hypoxemic respiratory failure: a systematic review and meta-analysis
Source: Syst Rev. 2017 Oct 16;6:202. doi: 10.1186/s13643-017-0593-5 (PMC5644261; doi:10.1186/s13643-017-0593-5)
Supplement: Supplementary file 4 — GRADE summary of evidence table. (DOCX 13 kb) [file 13643_2017_593_MOESM4_ESM.docx]

Appendix 4 – GRADE Summary of Evidence Table

Author(s): Leeies MA, Zarychanski R.

Date: June 10, 2016

Question: High-flow nasal cannulae compared to Active comparator for patients with acute hypoxemic respiratory failure

Setting: Emergency Departments and Intensive Care Units

| Quality assessment | | | | | | | № of patients | | Effect | | Quality | Importance |
| --- | --- | --- | --- | --- | --- | --- | --- | --- | --- | --- | --- | --- |
| № of studies | Study design | Risk of bias | Inconsistency | Indirectness | Imprecision | Other considerations | High-flow nasal cannulae | Active comparator | Relative (95% CI) | Absolute (95% CI) |  |  |
| Mortality | | | | | | | | | | | | |
| 5 | randomised trials | serious ^1^ | serious ^2^ | not serious ^2^ | serious ^3^ | none | 111/775 (14.3%) | 132/854 (15.5%) | RR 1.01 (0.69 to 1.48) | 2 more per 1,000 (from 48 fewer to 74 more) | ⨁◯◯◯ VERY LOW | CRITICAL |

**CI:** Confidence interval; **RR:** Risk ratio

1. No trials were blinded so all were deemed at high risk of bias.
2. There was significant variation in the duration of study treatments with questionable post-randomization treatment differences. The true impact on mortality in 2/5 studies is questioned.
3. Confidence intervals include credible estimates of both benefit and harm.
