# Supplementary material for: High-flow oxygen via nasal cannulae in patients with acute hypoxemic respiratory failure: a systematic review and meta-analysis
Source: Syst Rev. 2017 Oct 16;6:202. doi: 10.1186/s13643-017-0593-5 (PMC5644261; doi:10.1186/s13643-017-0593-5)

Appendix 5 – Mortality: Subgroup Analysis

5.1 – Mortality: Subgroup by Comparator


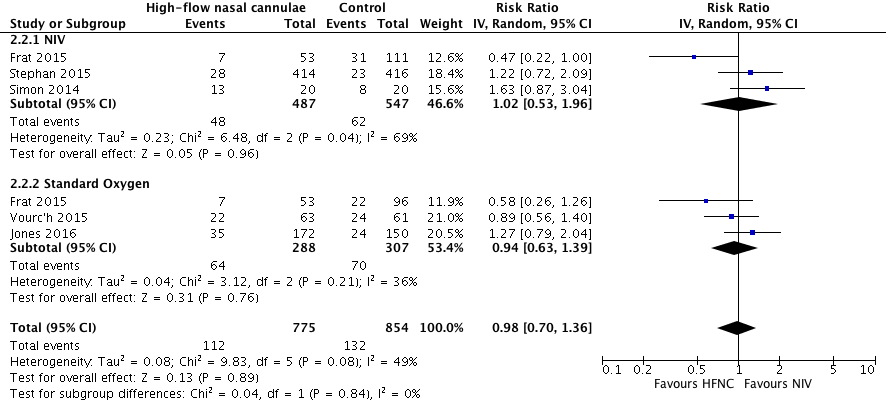


5.2 – Mortality: Subgroup by Population


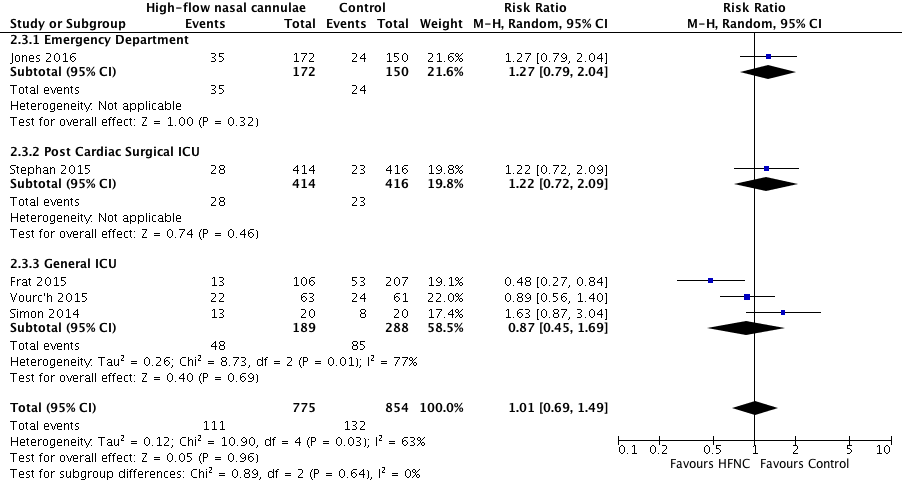


5.3 – Mortality: Subgroup by Duration of Therapy


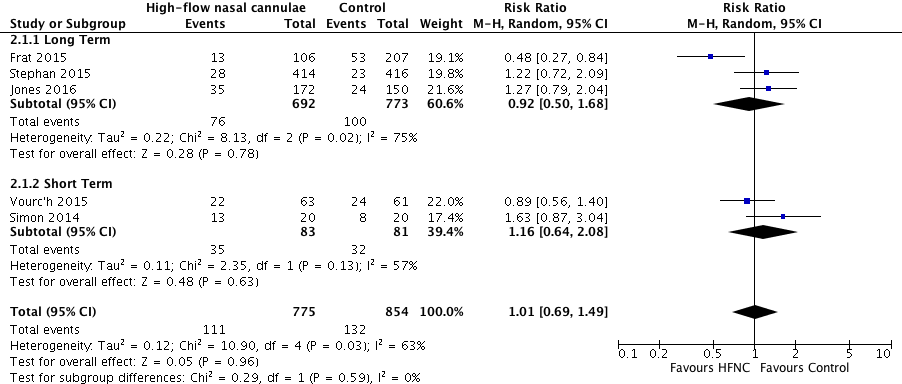

Supplement: Supplementary file 5 — Mortality: subgroup analysis. (DOCX 4447 kb) [file 13643_2017_593_MOESM5_ESM.docx]
