# Supplementary figures and images for: High-flow oxygen via nasal cannulae in patients with acute hypoxemic respiratory failure: a systematic review and meta-analysis
Source: Syst Rev. 2017 Oct 16;6:202. doi: 10.1186/s13643-017-0593-5 (PMC5644261; doi:10.1186/s13643-017-0593-5)

Appendix 6 – Trial Sequential Analysis for Mortality


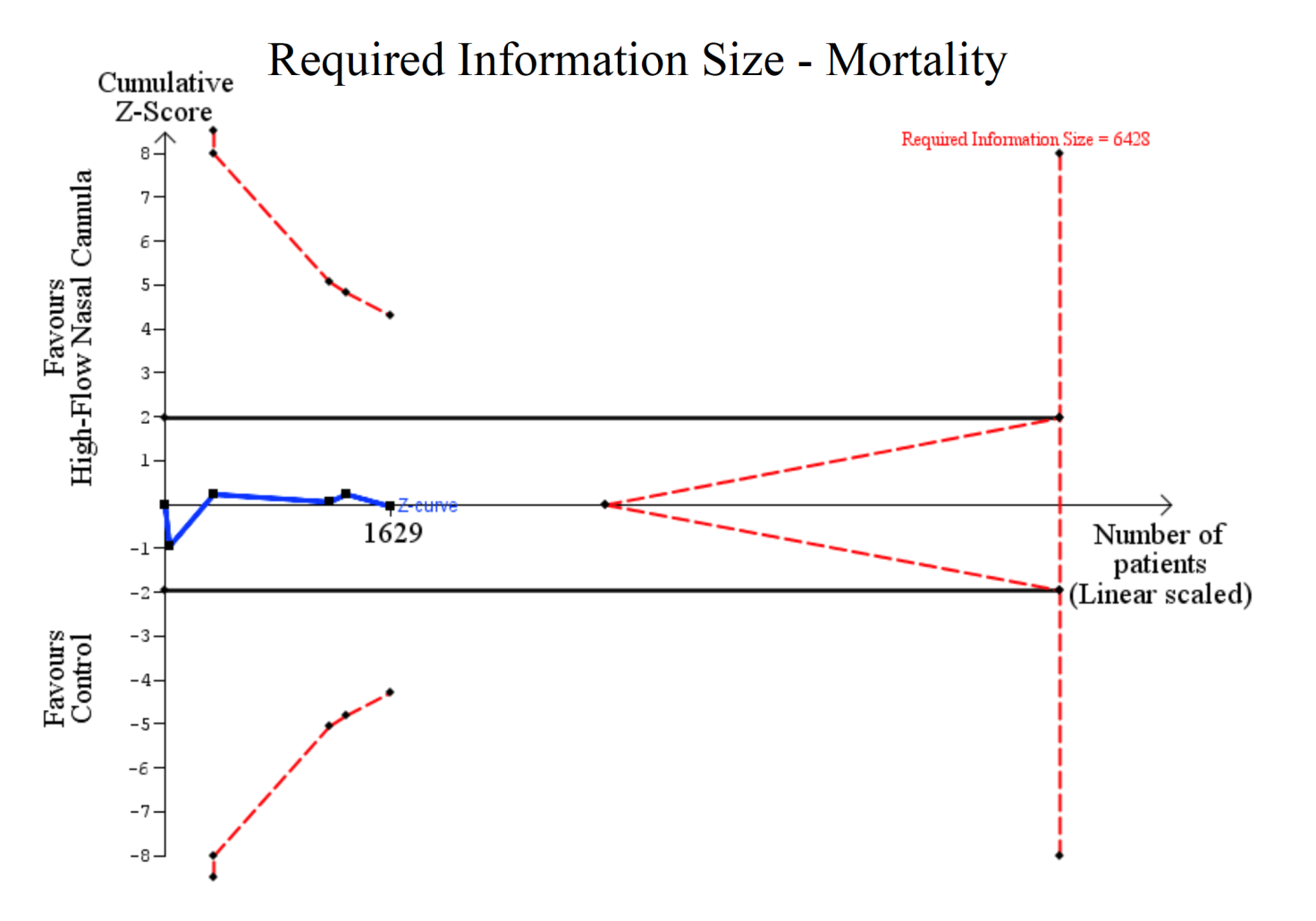

Supplement: Supplementary file 6 — Trial sequential analysis for mortality. (DOCX 257 kb) [file 13643_2017_593_MOESM6_ESM.docx]
